# Dual-Mechanism Study of Metal-Free g-C_3_N_4_ Catalysts for Advanced Oxidation Under Non-Photocatalytic Conditions

**DOI:** 10.3390/molecules30020247

**Published:** 2025-01-10

**Authors:** Zixu Yang, Yang Sun, Weizhi Wang, Xiaohan Yuan, Pengfei Tian, Jing Xu

**Affiliations:** 1 State Key Laboratory of Chemical Engineering, East China University of Science and Technology, Shanghai 200237, China; zixu.yang@ecust.edu.cn (Z.Y.);; 2 Key Laboratory of Pressure Systems and Safety (Ministry of Education), School of Mechanical and Power Engineering, East China University of Science and Technology, Shanghai 200237, China

**Keywords:** metal-free, light-independent, nonradical degradation, advanced oxidation processes, g-C_3_N_4_

## Abstract

Metal-free materials have been proved to be promising replacements of traditional metal-based catalysts for advanced oxidation reactions. Carbon nitride was found to be able to activate H_2_O_2_ and generate hydroxyl radicals (•OH). Nevertheless, the performance of carbon nitride is highly dependent on an external light source. In this work, we report a light-independent, metal-free catalyst based on g-C_3_N_4_ prepared using a facile calcination method. It is revealed that two reaction pathways, a radical (•OH) one and a nonradical (H_2_O_2_) one, coexist in organics oxidation on g-C_3_N_4_. The dominant reaction pathway is dependent on the condensation temperature of UCN. In addition, this g-C_3_N_4_ exhibited excellent stability after being recycled and reused for five cycles. The findings in this work can be used for the design of efficient and robust metal-free catalysts with both superior catalytic performance and high stability for various heterogeneous catalytic processes.

## 1. Introduction

The development of high-efficiency catalysts for the activation of H_2_O_2_ to degrade organic contaminants is one of the research hotpots of environmental science [1,2,3]. An optimal catalyst should combine an excellent ability to activate the H_2_O_2_ molecules, high stability, and avoidance of secondary pollution (heavy metal, extreme pH). In addition, it should be non-toxic, low-cost, and easy to synthesize. Over the past decades, various metal catalysts have been developed for H_2_O_2_ activation to remove organic pollutants. In particular, it has been found that a Cu-based catalyst is more advantageous for practical application due to the avoidance of producing a large amount of undesired iron sludge in the effluent, facile separation of the catalyst, and a broad operating pH range compared with conventional Fenton catalyst (Fe^2+^). To further improve the catalytic performance of mono-Cu catalysts, a second metal, such as iron [4], nickel [5], cobalt [6,7], or manganese [8], is usually added. However, heavy metal leaching is still inevitable during the degradation process. Therefore, metal-free catalysts were proposed to solve this problem [9,10]. Lyu et al. suggested that reduced graphene oxide nanosheets could be utilized to efficiently remove 2-chlorophenol and bisphenol A [11]. Nonetheless, the preparation process of this kind of material is complex. In recent years, carbon nitride (C_3_N_4_) with a defect-rich topology was found to be able to activate H_2_O_2_ and generate hydroxyl radicals (•OH) under the condition of light [12,13]. Recent studies have increasingly focused on metal-doped graphitic materials that can generate reactive radicals without relying on light irradiation. This emerging direction addresses the limitations of conventional photocatalysis by eliminating the need for external light sources, thus reducing both energy consumption and operational costs. For instance, research on Fe/Ni/Pd-doped g-C_3_N_4_ catalysts has demonstrated the in situ generation of H_2_O_2_ and subsequent production of reactive oxygen species (•OH, •O_2_^−^) under dark conditions, leading to effective degradation of refractory organics with high catalyst stability [14]. Similarly, oxygen-doped g-C_3_N_4_ with abundant defect sites can activate H_2_O_2_ in the absence of light to achieve enhanced degradation performance while maintaining a metal-free, stable structure [15]. In other work, the construction of dual-active sites by anchoring Fe^0^ onto N-rich carbon supports or precisely tuning the nitrogen configurations in graphene-based materials has proven effective for non-photoactivated PMS activation, yielding a variety of radicals (•OH, ^1^O_2_) and enabling broad substrate applicability [16,17]. Ge et al. found that g-C_3_N_4_/MgO nanosheets could activate H_2_O_2_ and generate •OH without light irradiation; the activity of this catalyst decreased constantly with the leaching of magnesium [18]. Collectively, these investigations highlight that through judicious material design—such as introducing specific dopants, tailoring defect sites, and controlling nitrogen configurations—catalysts can facilitate robust radical generation under dark conditions. 

To the best of our knowledge, g-C_3_N_4_ is the most stable allotrope of carbon nitride that could be synthesized through thermal condensation of nitrogen-rich precursors, such as melamine [19,20], urea [21], cyanamide [22], or dicyandiamide [12,23]. Various synthesis routes have been developed to tailor the properties of g-C_3_N_4_, including thermal polymerization, chemical vapor deposition (CVD), solvothermal/hydrothermal methods, and template-assisted approaches [24,25,26]. Among these methods, thermal polymerization of low-cost precursors is one of the most widely employed because of its simplicity, cost-effectiveness, and scalability. This route typically involves heating the precursor at moderate temperatures (500–600 °C), allowing the formation of layered g-C_3_N_4_ structures suitable for a range of applications. While CVD enables precise control over morphology, it requires specialized equipment and higher costs. Solvothermal/hydrothermal methods and template-assisted synthesis allow for detailed morphological tuning, but these approaches tend to introduce additional complexity and often require multiple synthesis steps or templates. Doping and co-doping strategies further enhance photocatalytic performance, but also increase synthetic complexity and cost.

In contrast, the thermal polymerization (calcination) method used in this study offers a straightforward, direct route to tune the polymerization degree of g-C_3_N_4_ simply by adjusting the condensation temperature. This approach enables us to systematically investigate the structure–activity relationship without introducing extra complexity or expensive metal components. 

The latter two kinds of precursors are virulent and expensive in comparison to the former two. Furthermore, the catalytic performance of carbon nitride is directly affected by the selection of the precursor. Our preliminary results suggested that the melamine-derived catalyst achieved 47.3% MB removal within 30 min, the urea-derived catalyst achieved 71.6%, and the dicyandiamide-derived catalyst achieved 21.8% under identical reaction conditions (Figure S1). In this work, we report a light-independent, metal-free and highly stable Fenton-like catalyst based on g-C_3_N_4_ prepared using a facile calcination method. This light-independent g-C_3_N_4_ catalyst has never been reported before as far as we know. On the basis of the cost of raw materials and catalytic activity, C_3_N_4_ synthesized from urea was studied in this work. Unlike conventional g-C_3_N_4_-based catalysts that rely on photoexcitation to generate reactive radicals, the g-C_3_N_4_ catalyst reported in this study is capable of activating H_2_O_2_ and degrading organic pollutants under completely dark conditions. This represents a significant departure from previously reported systems, where external light sources were generally essential. The ability to operate without light irradiation not only expands the practical applicability of g-C_3_N_4_-based advanced oxidation processes to settings where continuous illumination is impractical or costly, but also greatly reduces energy consumption and simplifies the reactor design. In other words, this light-independent process can substantially lower operational costs and complexity, facilitating industrial-scale wastewater treatment and environmental remediation. Furthermore, by remaining metal-free, our catalyst avoids the heavy metal leaching problems associated with metal-based Fenton-like catalysts, thereby enhancing its long-term stability and environmental compatibility.

## 2. Results and Discussion

### 2.1. Morphology and Crystal Structure

**Scheme 1:**
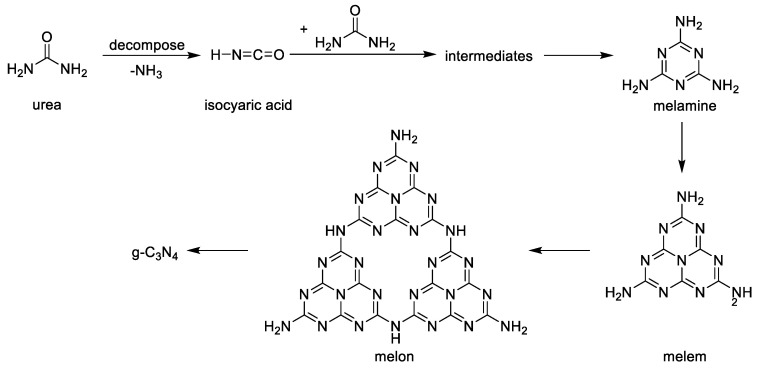
The formation mechanism of g-C_3_N_4_ synthesized by urea.

Generally, the crystal structures of g-C_3_N_4_ can be tuned by using different condensation temperatures. Xu et al. explained two different structures of g-C_3_N_4_ through thermal condensation of urea. During the heating process, the transformation of urea could be summarized as follows: urea → ammonia and isocyanic acid → cyanuric acid and other intermediates → melamine → melem and melon (Scheme 1). It was observed that the g-C_3_N_4_ with a planar structure (Figure S2a) could be formed when melem was fully condensed, while the g-C_3_N_4_ with a linear structure (Figure S2b) could be formed when melem was partially condensed to form NH bridges [27].

**Figure 1:**
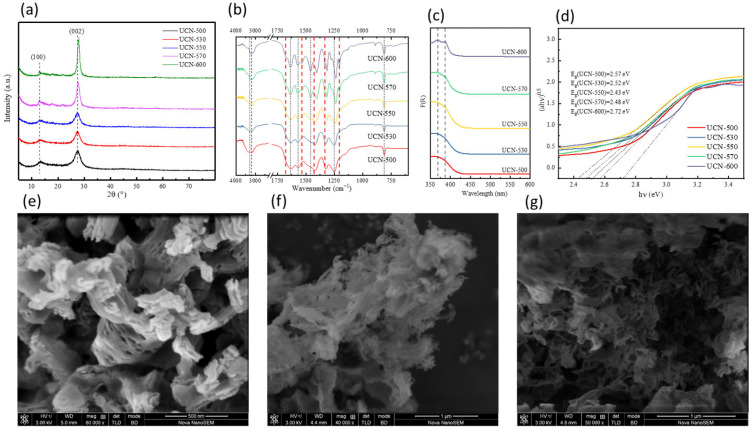
(**a**) XRD patterns, (**b**) FTIR spectra and (**c**) UV-Vis DRS spectra of as-prepared UCN-T. (**d**) The plots of (ahν)^1/2^ vs. hν. (**e**) SEM images of UCN-550. (**f**) SEM images of UCN-570. (**g**) SEM images of UCN-600. Characteristic peaks are designated using dashed lines.

Figure 1a shows the XRD patterns of as-prepared samples. Two peaks at ca. 27.6° and 13.1° for all the samples could be ascribed to the (002) interplanar stacking of aromatic systems such as triazine or heptazine rings, and (110) in-plane structural packing motif stacking, respectively [27,28]. The intensities of both peaks became stronger and narrower with increasing condensation temperature, indicating that the crystallinity of UCN-T samples increases with the rise in temperature. In addition, the (002) peak shifted toward a higher diffraction angle from 27.5° to 27.8°, and the corresponding interlayer distance decreased from 0.324 nm to 0.321 nm, suggesting the improvement in the interlayer stacking order of UCN-T and the tendency towards structural stability. In the meantime, the (110) peak shifted to lower diffraction angle from 13.1° to 12.8°, indicating that the corresponding hole-to-hole distance of nitride pores increased from 0.675 nm to 0.691 nm, suggesting that the polymerization degree became higher.

The functional groups can be further confirmed by FTIR spectroscopy in Figure 1b. The broad band between 3500 and 3000 cm^−1^ can be ascribed to the N-H stretches of -NH and the O-H stretches of the physically adsorbed water [29]. Additionally, the characteristics of the out-of-plane bending vibration of triazine or heptazine rings at 809 cm^−1^ was also observed. The peaks in the range of 1700 and 1500 cm^−1^ are characteristic of C=N, while the peaks in the region of 1500–1200 cm^−1^ can be assigned to C-N in heterocycles (either trigonal C-N(-C)-C or bridging C-NH-C units), which are usually related with skeletal stretching vibrations of aromatic rings [30]. Interestingly, the intensities of the peaks at 1200, 1334, 1427, 1538, and 1683 cm^−1^ become stronger (red dash line) with the rising condensation temperature. This might be caused by a more ordered packing of hydrogen-bond-cohered long strands of polymeric melon units [31], which further confirmed the increase in polymerization degree of samples.

Figure 1c shows the UV-Vis DRS spectra of UCN samples synthesized at different temperatures. The peaks at 367 nm and 385 nm ascribed to the π → π* conjugation effect of aromatics rings became stronger when temperature was higher, indicating a higher polymerization degree. Meanwhile, the absorption edges were varied by changing the temperature (Figure 1d). When the temperature increased, the absorption edge first shifted up until the temperature reached 550 °C; then, the absorption edge decreased. The band gap energy (E_g_) can be estimated from the intercept of the tangents to the plots of (ahν)^0.5^ vs. photon energy. The calculated E_g_ value is around 2.7 eV, which is consistent with previous reports [32]. When the calcination temperature increased from 500 to 550 °C, the E_g_ value decreased slightly from 2.57 to 2.43 eV. The bathochromic shift of the band gap can be ascribed to a stronger overlap of molecular orbitals due to the reinforced aromatic sheet stacking, because the packing of tri-striazine units that are connected via Van der Waals interaction were enhanced along with the elevated condensation temperature [33]. When the temperature was further increased to 600 °C, E_g_ was increased from 2.43 to 2.72 eV. The hypsochromic shift of the band gap resulted from the quantum confinement effects induced by the transformation of bulk g-C_3_N_4_ into nanoscale sheets with reduced size and thickness [13].

The morphologic variance of different UCN samples mentioned above was intuitively investigated with scanning electron microscopy (SEM). UCN-550 showed a layer structure with an obvious pore structure, and the layers were thicker than those of UCN-570 and UCN-600. Moreover, the edges of the layers were neat when the condensation temperature was 550 °C. However, with the increase in temperature, the layers gradually became thinner and the edges were ragged as a result of minimizing the surface energy of the sheet [31]. When the urea was heated at 600 °C, the sample appeared as loose and soft agglomerates with a flocculent structure. Further close observation of a SEM image indicates the presence of a porous layer on the surface.

### 2.2. Catalytic Performance

**Figure 2:**
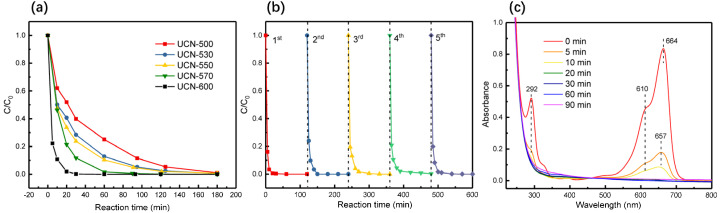
(**a**) MB removal efficiencies over different catalysts. (**b**) Stability of UCN-600 for MB degradation. (**c**) The UV-Vis absorption spectra of MB solution during the reaction. (Reaction conditions: 50 °C, 1000 ppm H_2_O_2_, 0.5 g·L^−1^ catalyst.).

The catalytic performances of the metal-free UCN samples were evaluated in catalytic activation of H_2_O_2_ for methyl blue (MB) degradation in water solutions. The as-prepared UCN and H_2_O_2_ were simultaneously added into 5 ppm MB solution in the dark at 50 °C to initiate the reaction, and the catalytic activity was estimated via analyzing the concentration of MB at different reaction times by UV-Vis spectroscopy (664 nm). In addition, a blank reaction was also performed to exclude the adsorption effect. As shown in Figure 2a, the removal rate of MB increased with the increasing condensation temperature. The removal efficiency of UCN-500 was 38.1% in 10 min, and it was increased to 49.9%, 52.3%, 53.8%, and 99.3% when the condensation temperature was 530, 550, 570, and 600 °C, respectively. UCN-600 exhibited 77.8% removal efficiency in only 5 min, while its adsorption efficiency was 42.3% at the same time, indicating that still more than 35% of removal efficiency can be attributed to the degradation effect. Furthermore, UCN-600 was recycled and reused after each experiment to evaluate its stability, and it retained a high removal efficiency after five cycles, manifesting its excellent stability (Figure 2b). This can be explained by the durability of g-C_3_N_4_. For comparison, we previously evaluated Fe-Cu bimetallic and Cu-based catalysts under similar experimental conditions, achieving complete MB degradation within 20 min. Remarkably, the metal-free g-C_3_N_4_ catalyst in this study demonstrated a comparable degradation efficiency, also reaching nearly 100% removal within the same time-on-stream. The g-C_3_N_4_ catalyst also exhibited excellent reusability owing to its chemical stability, and this was confirmed by the unchanged IR spectra [34].

Furthermore, in order to gain insight into the MB degradation, the UV-Vis spectra of MB solution were recorded at different reaction times (Figure 2c). A main band with maximum absorption at 664 nm and a small shoulder at 610 nm was located in the visible region, and a band at 292 nm was found in the ultraviolet region. The absorbance at 664 nm can be assigned to the conjugation effect of two dimethylamine-substituted aromatic rings through sulfur and nitrogen, while the shoulder at 610 nm can be attributed to the dye dimmer; the absorption bands in the ultraviolet region are caused by the substituted benzenes rings. It was clearly observed that the intensity of the peaks decreased rapidly, and the peaks at 664 nm slightly shifted to 657 nm during the reaction, indicating the formation of the demethylated dyes [35].

### 2.3. Oxidative Degradation Pathway

It already known that the primary oxidant in light-driven photo-Fenton systems catalyzed by carbon nitride is •OH, and sometimes •OOH [36,37]. The •OH is generated via the direct hole oxidation or multi-step reduction of dissolved O_2_ induced by photogenerated electrons (Equations (1) and (2)), so it is not difficult to notice that the generation of •OH under light irradiation relies on light to a large extent [19].

O_2_ + e^−^ → •O_2_ (1)

•O_2_ + 2e + 2H^+^ → H_2_O_2_ (2)

H_2_O_2_ + e → •OH + OH^−^ (3)

However, all of the degradation process in this work was carried out in dark, so the radical mechanism above is no longer appropriate. Therefore, the reactive oxidant in our system needs to be reconsidered.

**Figure 3:**
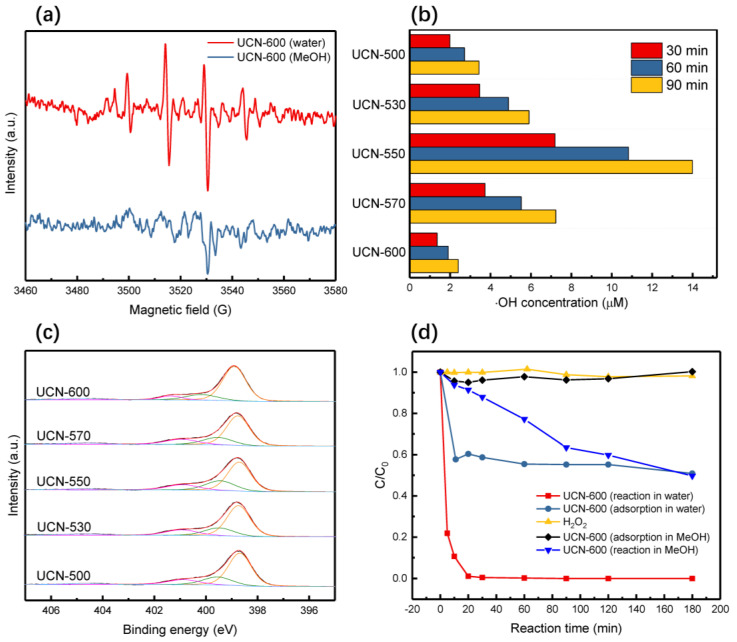
(**a**) BMPO spin-trapped EPR of UCN-600 in different solvents. (**b**) The concentration of generated •OH using different catalysts. (Reaction conditions: 50 °C, 1000 ppm H_2_O_2_, 0.5 g/L UCN-600, 1500 ppm benzoic acid.) (**c**) N 1s XPS spectra of the UCN samples. The black curves represent the original XPS signals, and the colored curves represent the fitted curves. (**d**) MB removal efficiencies in different solvents over UCN-600.

Here, we first employed electron paramagnetic resonance (EPR) to probe the generation of reactive radicals using 5,5-dimethyl-1-pyrroline (DMPO) as a radical spin-trapping agent. As illustrated in Figure 3a (red line), the characteristic quartet peaks of DMPO-•OH adduct with an intensity ratio of ca. 1:2:2:1 can be observed, indicating the generation of •OH in aqueous solution. After quantifying the concentration of •OH, we found that the cumulative concentration of •OH first increased with condensation temperature and reached its maximum value at 550 °C, and then gradually decreased.

According to the generation mechanism of •OH mentioned before, the production of •OH is considered to be related with the electron transfer between catalyst and H_2_O_2_. In addition, the vacancies on the catalyst surface, which can modify the electronic structure and often be considered as specific reaction sites for reactant molecules, have been proven to be related to catalytic performance [7]. Therefore, N 1s XPS spectra were measured (Figure 3c and Figure S2). The three peaks at 398.7, 399.5, and 400.5 eV are attributed to the sp^2^-bonded N (C-N=C, N_2c_), the sp^2^-hybridized N bonded to three atoms (N-C_3_ or C_2_-HN, N_3c_), and amino groups (C-N-H), respectively. In addition, the peak at 404.6 eV is attributed to the charging effects or positive charge localization in the heterocycles [33]. The ratios of atomic concentrations of C, N, and O in different UCNs are listed in Table 1. The atomic molar ratio of C to N decreased from 0.75 to 0.66 with the condensation temperature increasing from 500 °C to 600 °C, consistent with previous work [13]. The peak ratio of N_2c_ to N_3c_ was also calculated, and the value was first decreased from 3.2 to 2.4 when the condensation temperature was raised from 500 °C to 550 °C; then, it increased from 2.4 to 5.8 when the condensation temperature was further increased to 600 °C. A possible explanation for the decreasing N_2c_ peak area between 500 °C and 550 °C is that the partial condensation brought about more C_2_-HN structures and, subsequently, more N_3c_. Nevertheless, further higher condensation temperatures (570 °C and 600 °C) promoted further condensation of terminal amino groups, causing a higher polymerization degree and producing more heptazine rings, subsequently increasing the percentage of N_2c_ [29]. These structural evolutions obtained through XPS are consistent with the results from XRD, FTIR, and UV-Vis DRS. Nitrogen vacancies mainly locate at the N_2c_ lattice sites during the condensation [23], so the concentration of nitrogen vacancies reached its maximum at 550 °C. In combination with the •OH formation in Figure 3b and XPS results, it can be deduced that the •OH is generated through positively charged C caused by nitrogen vacancies on g-C_3_N_4_, so the degradation performance was positively correlated with the concentration of nitrogen vacancies.

If •OH was the only main reactive oxygen species, catalytic performance should also be optimal at 550 °C, but it is easy to realize from Figure 2a that this is not the case. This phenomenon indicates that there may be another oxidation pathway in addition to the •OH oxidation pathway. As we all know, the degradation would be significantly suppressed in alcohol solvents if •OH is the key species for the organic degradation. Thus, methanol was applied as the reaction solvent instead of water to scavenge •OH generated in the activation process to confirm this. It can be found in Figure 3d that 40.0% of MB still could be gradually removed in methanol, even though the removal efficiency was lower than that in aqueous solution, proving that •OH is not the only active oxygen species in this system. Due to the stable property of •OOH in methanol and its relatively strong oxidation property, EPR was used to detect the presence of •OOH in the methanol reaction system. Almost no signal was observed in methanol solution (blue line in Figure 3a), and the degradation effect caused by •OOH could be excluded. Meanwhile, as illustrated in Figure 3c, the concentration of MB was unchanged in the degradation system without UCN-600, so the degradation effect caused by H_2_O_2_ could be also excluded. Therefore, it can be speculated that there may be two degradation pathways in the aqueous solution system: (i) the free radical oxidation pathway of •OH (as the main active oxygen radical), and (ii) nonradical oxidation pathway. Furthermore, the nonradical oxidation pathway may dominate the degradation process when the condensation temperature is higher than 550 °C.

Novel nonradical reactions have attracted great attention in the past five years, and the reaction mechanisms of different materials are different. An important characteristic of nonradical oxidation that distinguishes it from radical-based oxidation is that the organics are supposed to be oxidized on the surface of catalysts instead of in the bulk solution [38]. These nonradical AOPs (NR-AOPs) are increasingly recognized for their high selectivity, wider pH tolerance, and stronger resistance to interference from background constituents [39]. Unlike free radical-driven processes, NR-AOPs are primarily dominated by interfacial catalytic activation of oxidants (H_2_O_2_, PMS, PDS, etc.) on catalyst surfaces, leading to the formation of nonradical species such as singlet oxygen (^1^O_2_), high-valent metal species (e.g., Fe(IV), Co(IV), Cu(III)), surface-bound reactive complexes, and direct electron-transfer pathways [40,41,42,43]. Accordingly, the oxidants should be activated on the surface without releasing the free radicals into the solution. Tang’s group proved that the pre-adsorption of the phenolics was a key step in this peroxydisulfate (PDS) system dominated by graphene surface-confined sulfate radicals [44]. At present, there are still few studies on the nonradical oxidation pathway in the H_2_O_2_ system.

A nonradical pathway accompanied by radical generation (•OH and SO_4_•^−^) in phenol oxidation was also discovered upon N-doped nanocarbon by Wang et al. [44]. They thought the C atoms could be activated by the doped N atoms of higher electronegativity to form positively charged carbon domains, which have the capacity to interact with the O-O bond of peroxymonosulfate (PMS) to generate a highly reactive complex and decompose organics via direct electron transfer [45]. Moreover, it has been widely accepted that the carbon atoms at the edging sites remained sp^2^ conjugated with unpaired electrons in a ‘localized state’, which were able to strongly interact with the small molecules to form an attached complex [38].

Combining the opposite trend of •OH formation, MB degradation, and XPS results, a possible H_2_O_2_ activation and MB degradation mechanism could be proposed. After the condensation temperature was over 550 °C, a nonradical pathway may dominate the degradation process. In this nonradical process, the H_2_O_2_ might be first bonded with the sp^2^-hybridized system (C-N_2c_=C) and then generate surface-bounded •OH. The highly covalent π electrons could activate the O-O bond in H_2_O_2_, and the interaction strength could be predicted by the N_2c_ content (Table 1). The adsorption–decomposition of MB on UCN through the nonradical oxidation pathway in the presence of H_2_O_2_ was proposed as follows: (1) MB in solution was adsorbed on the surface of UCN due to the strong π-π interaction; (2) the H_2_O_2_ molecules were activated, and surface-bound •OH (UCN≡•OH) was produced; (3) the adsorbed MB was in situ oxidized by the UCN≡•OH, releasing the preoccupied sites as free sites; and (4) the released free sites on UCN allowed a new adsorption of MB in solution, and then an adsorption–degradation cycle started again. The above-mentioned cycles were repeated continuously until all the added MB was degraded.

**Figure 4:**
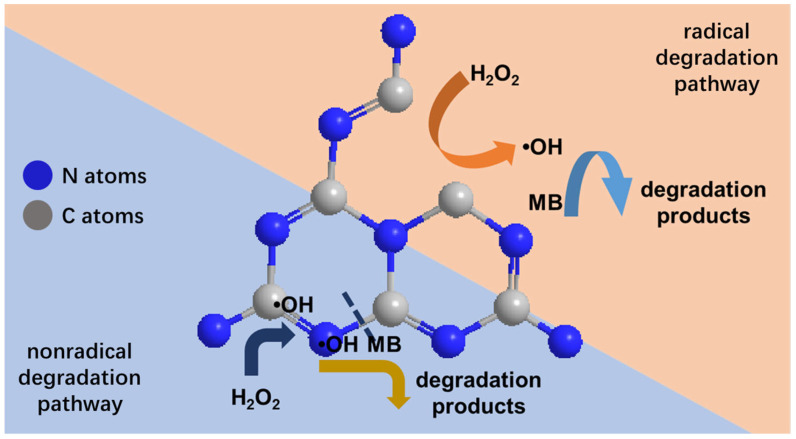
Degradation mechanism over UCN catalyst in the absence of light.

As illustrated in Figure 4, both radical and nonradical pathways were proposed to contribute to the MB degradation on UCN with H_2_O_2_ activation, and the dominant one could be determined by the condensation temperature of UCN. When the condensation temperature is below 550 °C, a radical degradation pathway is dominant, and N vacancies play the key role on MB degradation; when the condensation temperature is above 550 °C, a nonradical degradation pathway is dominant, and the presence of the sp^2^-hybridized system of C-N_2c_=C might affect the generation of UCN≡•OH and the oxidation of MB.

## 3. Experimental Methods

### 3.1. Materials

Carbon nitrides were prepared by heating 10 g of precursor (urea, melamine, or dicyandiamide) in a 50 mL alumina crucible in a muffle furnace. Considering the differences in performance and structure of the catalysts caused by different condensation temperature [22,27], the crucible was covered, heated at a ramping rate of 10 °C/min, and kept at the terminal calcination temperature (T = 500, 530, 550, 570, 600 °C) for 2 h. After cooling, the yellowish powders were ground and collected. Given the superior catalytic activity of the urea-derived catalyst, along with the low cost and easy availability of urea, we selected urea as the precursor for all subsequent experiments. Thus, the catalysts discussed in the main text are denoted as UCN-T (where T represents the calcination temperature), indicating that they are exclusively derived from urea. 

### 3.2. Characterization

The X-ray powder diffraction (XRD) pattern was measured with a Bruker D8 Advance diffractometer (Bruker Corp., Billerica, MA, USA) using a CuKa ray source (λ = 0.154 nm) with accelerated voltage of 40 kV and a 40 mA detector current in an angular range of 10−80°. Fourier transform infrared (FT-IR) spectra were examined in a PerkinElmer spectrum 100 (PerkinElmer Inc., Waltham, MA, USA). Samples were mixed with KBr powder (spectral purity) at a sample/KBr weight ratio of 1/750. The mixture was then ground to 100 mesh size and pressed into a lucid disk, which was then directly placed into the IR spectrometer for measurement within the frequency range of 4000–450 cm^−1^. UV-Vis DRS spectra were collected using a Lambda 750 UV-Vis-NIR spectrometer (PerkinElmer Inc., Waltham, MA, USA) equipped with a diffuse reflectance spectroscopy (DRS) accessory. The spectra were collected between 350 and 600 nm with an interval of 1 nm using BaSO_4_ as a reference, and were transformed into the Kubelka–Munk function, F(R). XPS analysis was carried out on a Thermo Fisher Scientific ESCALAB 250Xi spectrometer (Thermo Fischer Scientific Inc., Waltham, MA, USA) with Al-Kα radiation (hν = 1486.6 eV, pass energy: 40.0 eV) as the X-ray source. To compensate for surface charge effects, all the binding energies (BEs) were calibrated using the C1s line at 284.80 eV.

An electron paramagnetic resonance (EPR) spectrometer (Bruker EMX-8/2.7C, Bruker Corp., Billerica, MA, USA) was employed to detect the •H radicals. The measurements were conducted under the following conditions: center field at 3520 G, scan width of 200 G, microwave frequency of 9.877 GHz, microwave power of 6.384 mW, scan time of 41.96 s, and a signal gain of 7.10 × 10^4^.

Prior to the EPR measurement, 50 mL of 1000 ppm H_2_O_2_ solution was adjusted to pH 2.7 and heated to 40 °C. Subsequently, 0.025 g of the catalyst was dispersed in this solution. After reacting for 5 min, a 1 mL sample was withdrawn, filtered, and mixed immediately with 1 mL of a 20 mM DMPO solution (spin-trapping agent). From this mixture, 20 μL was drawn into a syringe and then transferred into a capillary tube, which was placed inside a *φ*5 × 250 mm quartz EPR tube for analysis.

### 3.3. Catalytic Performance Evaluation

The catalytic activity of UCNs was evaluated for the degradation of methyl blue (MB). MB is a common textile dye that is chemically stable and frequently used as a probe molecule in advanced oxidation process studies due to its well-defined absorption spectra and easy quantification. Its molecular structure shares certain features (e.g., aromatic rings, functional groups) with a variety of other organic dyes and pollutants. Thus, the efficient removal of MB implies the potential applicability of our catalyst to a broader range of organic contaminants found in wastewater. A typical reaction was performed in a batch reactor containing 100 mL of 5 ppm MB, 0.5 g L^−1^ catalyst, and 1000 ppm H_2_O_2_ while the suspension was stirred at 50 °C in dark. At the given reaction time intervals, 3 mL supernatant solution was collected by filtration through a 0.22 μm Nafion membrane for the analysis of MB concentration. The concentration of MB was determined at its maximum absorption wavelength of 554 nm using an UV-Vis spectrophotometer. The copper leaching was measured by ICP-MS. The concentration of ·OH was measured indirectly using benzoic acid as a probe molecule.

To evaluate the reusability of the catalyst, the spent catalyst from each experiment was collected, washed with deionized water, dried, and then reloaded in the reactor for recycling tests under the same conditions.

The concentration of •OH was measured indirectly using benzoic acid as a probe molecule. The MB degradation process was monitored by a Lambda 750 UV-Vis-NIR spectrometer. The spectra were collected between 200 and 800 nm with an interval of 1 nm using water as a reference.

## 4. Conclusions

This study demonstrates, for the first time, that a urea-derived g-C_3_N_4_ catalyst can efficiently activate H_2_O_2_ and degrade organic pollutants without any light irradiation. By adjusting the calcination temperature, we revealed the coexistence and switchable dominance of radical and nonradical pathways, enabling fine-tuned catalytic performance without the need for metals or complex doping. In particular, UCN-600 presented an extraordinarily high catalytic stability for MB removal by H_2_O_2_ activation in the absence of light. Since UCN indeed is a kind of photocatalytic material, the coexistence of different degradation mechanisms in visible light should be investigated in a future study. This study provides insights into a novel view of the activation of H_2_O_2_ over metal-free catalysts, and the findings can be used for the design of efficient and robust metal-free catalysts with high stability for organic contaminant degradation with heavy metal pollution. Unlike conventional g-C_3_N_4_-based catalysts that require photoexcitation, our catalyst achieves efficient H_2_O_2_ activation and pollutant degradation in complete darkness. This eliminates the need for continuous illumination, significantly lowering energy consumption and equipment costs. Moreover, the metal-free nature of this system prevents issues associated with heavy metal leaching. Together, these attributes mark a substantial step forward in developing sustainable, cost-effective, and easily scalable advanced oxidation technologies.

## Figures and Tables

**Table 1:** XPS parameters of different UCN samples.

Sample	Atomic Concentration				N_2c_/N_3c_	The Peak Area Ratio of N_2c_
	N%	C%	O%	C/N		
UCN-500	56.12	42.02	1.85	0.75	3.2	0.64
UCN-530	58.35	40.08	1.57	0.69	3.1	0.61
UCN-550	58.52	39.92	1.57	0.68	2.4	0.58
UCN-570	58.82	39.63	1.55	0.67	3.2	0.62
UCN-600	60.03	39.97	0.00	0.66	5.8	0.70

## Data Availability

Data are contained within the article.

## Supplementary Materials

The following supporting information can be downloaded at: https://www.mdpi.com/article/10.3390/molecules30020247/s1, Figure S1. Degradation performance of g-C_3_N_4_ prepared with different precursor. (calcination program: 550 °C for 2 h; degradation condition: 5 ppm MB, 1000 ppm H_2_O_2_, 50 °C, 0.5 g L^−1^ catalyst); Figure S2. The molecular structures of g-C_3_N_4_ of different polymerization degree (a) partial condensation. (b) full condensation.

## References

[1] Yang X.J., Xu X.M., Xu J., Han Y.F. (2013). Iron oxychloride (FeOCl): An efficient Fenton-like catalyst for producing hydroxyl radicals in degradation of organic contaminants. J. Am. Chem. Soc..

[2] Li X., Wang B., Cao Y., Zhao S., Wang H., Feng X., Zhou J., Ma X. (2019). Water Contaminant Elimination Based on Metal–Organic Frameworks and Perspective on Their Industrial Applications. ACS Sustain. Chem. Eng..

[3] Sun Y., Tian P., Ding D., Yang Z., Wang W., Xin H., Xu J., Han Y.F. (2019). Revealing the active species of Cu-based catalysts for heterogeneous Fenton reaction. Appl. Catal. B Environ..

[4] Lei Y., Chen C.S., Tu Y.J., Huang Y.H., Zhang H. (2015). Heterogeneous degradation of organic pollutants by persulfate activated by CuO-Fe3O4: Mechanism, stability, and effects of pH and bicarbonate ions. Environ. Sci. Technol..

[5] Zhou S., Qian Z., Sun T., Xu J., Xia C. (2011). Catalytic wet peroxide oxidation of phenol over Cu-Ni-Al hydrotalcite. Appl. Clay Sci..

[6] Zhang M., Annamalai K.P., Liu L., Chen T., Gao J., Tao Y. (2017). Multiwalled carbon nanotube-supported CuCo_2_S_4_ as a heterogeneous Fenton-like catalyst with enhanced performance. RSC Adv..

[7] Guo X.X., Hu T.T., Meng B., Sun Y., Han Y.F. (2020). Catalytic degradation of anthraquinones-containing H_2_O_2_ production effluent over layered Co-Cu hydroxides: Defects facilitating hydroxyl radicals generation. Appl. Catal. B Environ..

[8] Zhang Y., Liu C., Xu B., Qi F., Chu W. (2016). Degradation of benzotriazole by a novel Fenton-like reaction with mesoporous Cu/MnO_2_: Combination of adsorption and catalysis oxidation. Appl. Catal. B Environ..

[9] Nieto-Juarez J.I., Pierzchła K., Sienkiewicz A., Kohn T. (2010). Inactivation of MS_2_ coliphage in Fenton and Fenton-like systems: Role of transition metals, hydrogen peroxide and sunlight. Environ. Sci. Technol..

[10] Hu P., Su H., Chen Z., Yu C., Li Q., Zhou B., Alvarez P.J.J., Long M. (2017). Selective degradation of organic pollutants using an efficient metal-free catalyst derived from carbonized polypyrrole via peroxymonosulfate activation. Environ. Sci. Technol..

[11] Lyu L., Yu G., Zhang L., Hu C., Sun Y. (2018). 4-phenoxyphenol-functionalized reduced graphene oxide nanosheets: A metal-free Fenton-like catalyst for pollutant destruction. Environ. Sci. Technol..

[12] Cui Y., Ding Z., Liu P., Antonietti M., Fu X., Wang X. (2012). Metal-free activation of H_2_O_2_ by g-C_3_N_4_ under visible light irradiation for the degradation of organic pollutants. Phys. Chem. Chem. Phys..

[13] Dong F., Wang Z., Li Y., Ho W.K., Lee S.C. (2014). Immobilization of polymeric g-C_3_N_4_ on structured ceramic foam for efficient visible light photocatalytic air purification with real indoor illumination. Environ. Sci. Technol..

[14] Wang L., Ma J., Guo Q., Liu L., Shou J., Sun A., Zhao L. (2021). In Situ Generation of Hydrogen Peroxide Using Polymetallic-Doped g-C_3_N_4_ for Pollutant Removal. Appl. Sci..

[15] Jiang T.-J., Luo C.-W., Xie C., Wei Y.-H., Li A. (2020). Synthesis of oxygen-doped graphitic carbon nitride and its application for the degradation of organic pollutants via dark Fenton-like reactions. RSC Adv..

[16] Zhang J., Gao W., Yue Y., Wang W., Tan F., Wang X., Qiao X., Wong P.K. (2021). Two-step assembly induced Fe^0^-anchored graphitic N-rich graphene with biactive centers for enhanced heterogeneous peroxymonosulfate activation. J. Mater. Chem. A.

[17] Zhang J., Liu H., Gao W., Cheng D., Tan F., Wang W., Wang X., Qiao X., Wong P.K., Yao Y. (2022). In situ zinc cyanamide coordination induced highly N-rich graphene for efficient peroxymonosulfate activation. J. Mater. Chem. A.

[18] Ge L., Peng Z., Wang W., Tan F., Wang X., Su B., Qiao X., Wong P.K. (2018). g-C_3_N_4_/MgO nanosheets: Light-independent, metal-poisoning-free catalysts for the activation of hydrogen peroxide to degrade organics. J. Mater. Chem. A.

[19] Yan S.C., Li Z.S., Zou Z.G. (2009). Photodegradation Performance of g-C_3_N_4_ fabricated by directly heating melamine. Langmuir.

[20] Zhong Y., Wang Z., Feng J., Yan S., Zhang H., Li Z., Zou Z. (2014). Improvement in photocatalytic H_2_ evolution over g-C_3_N_4_ prepared from protonated melamine. Appl. Surf. Sci..

[21] Zhang M., Xu J., Zong R., Zhu Y. (2014). Enhancement of visible light photocatalytic activities via porous structure of g-C_3_N_4_. Appl. Catal. B Environ..

[22] Wang X., Maeda K., Thomas A., Takanabe K., Xin G., Carlsson J.M., Domen K., Antonietti M. (2008). A metal-free polymeric photocatalyst for hydrogen production from water under visible light. Nat. Mater..

[23] Niu P., Liu G., Cheng H.M. (2012). Nitrogen vacancy-promoted photocatalytic activity of graphitic carbon nitride. J. Phys. Chem. C.

[24] Bhanderi D., Lakhani P., Modi C.K. (2024). Graphitic carbon nitride (g-C_3_N_4_) as an emerging photocatalyst for sustainable environmental applications: A comprehensive review. RSC Sustain..

[25] Huang Z., Shen M., Liu J., Ye J., Asefa T. (2021). Facile synthesis of an effective g-C_3_N_4_-based catalyst for advanced oxidation processes and degradation of organic compounds. J. Mater. Chem. A.

[26] Mo Z., She X., Li Y., Liu L., Huang L., Chen Z., Zhang Q., Xu H., Li H. (2015). Synthesis of g-C_3_N_4_ at different temperatures for superior visible/UV photocatalytic performance and photoelectrochemical sensing of MB solution. RSC Adv..

[27] Xu J., Li Y., Peng S., Lu G., Li S. (2013). Eosin Y-sensitized graphitic carbon nitride fabricated by heating urea for visible light photocatalytic hydrogen evolution: The effect of the pyrolysis temperature of urea. Phys. Chem. Chem. Phys..

[28] Yuan Y.P., Xu W.T., Yin L.S., Cao S.W., Liao Y.S., Tng Y.Q., Xue C. (2013). Large impact of heating time on physical properties and photocatalytic H_2_ production of g-C_3_N_4_ nanosheets synthesized through urea polymerization in Ar atmosphere. Int. J. Hydrogen Energy.

[29] Su Q., Sun J., Wang J., Yang Z., Cheng W., Zhang S. (2014). Urea-derived graphitic carbon nitride as an efficient heterogeneous catalyst for CO_2_ conversion into cyclic carbonates. Catal. Sci. Technol..

[30] Martha S., Nashim A., Parida K.M. (2013). Facile synthesis of highly active g-C_3_N_4_ for efficient hydrogen production under visible light. J. Mater. Chem. A.

[31] Niu P., Zhang L., Liu G., Cheng H.M. (2012). Graphene-like carbon nitride nanosheets for improved photocatalytic activities. Adv. Funct. Mater..

[32] Dong F., Wu L., Sun Y., Fu M., Wu Z., Lee S.C. (2011). Efficient synthesis of polymeric g-C_3_N_4_ layered materials as novel efficient visible light driven photocatalysts. J. Mater. Chem..

[33] Zhang G., Zhang J., Zhang M., Wang X. (2012). Polycondensation of thiourea into carbon nitride semiconductors as visible light photocatalysts. J. Mater. Chem..

[34] Wang Y., Wang X., Antonietti M. (2012). Polymeric graphitic carbon nitride as a heterogeneous organocatalyst: From photochemistry to multipurpose catalysis to sustainable chemistry. Angew. Chem. Int. Ed..

[35] Yogi C., Kojima K., Wada N., Tokumoto H., Takai T., Mizoguchi T., Tamiaki H. (2008). Photocatalytic degradation of methylene blue by TiO_2_ film and Au particles-TiO_2_ composite film. Thin Solid Films.

[36] Monteagudo J.M., Durán A., Martín I.S., Aguirre M. (2010). Effect of light source on the catalytic degradation of protocatechuic acid in a ferrioxalate-assisted photo-Fenton process. Appl. Catal. B Environ..

[37] Rahim Pouran S., Abdul Aziz A.R., Wan Daud W.M.A. (2015). Review on the main advances in photo-Fenton oxidation system for recalcitrant wastewaters. J. Ind. Eng. Chem..

[38] Duan X., Sun H., Shao Z., Wang S. (2018). Nonradical reactions in environmental remediation processes: Uncertainty and challenges. Appl. Catal. B Environ..

[39] Yu J., Tang L., Pang Y., Liang X., Lu Y., Feng H., Wang J., Deng L., Zou J., Zhu X. (2022). Non-radical oxidation in environmental catalysis: Recognition, identification, and perspectives. Chem. Eng. J..

[40] Li N., Lu W., Pei K., Yao Y., Chen W. (2015). Formation of high-valent cobalt-oxo phthalocyanine species in a cellulose matrix for eliminating organic pollutants. Appl. Catal. B Environ..

[41] Liang P., Zhang C., Duan X., Sun H., Liu S., Tade M.O., Wang S. (2017). An insight into metal organic framework derived N-doped graphene for the oxidative degradation of persistent contaminants: Formation mechanism and generation of singlet oxygen from peroxymonosulfate. Environ. Sci. Nano.

[42] Duan X., Sun H., Wang Y., Kang J., Wang S. (2015). N-Doping-Induced Nonradical Reaction on Single-Walled Carbon Nanotubes for Catalytic Phenol Oxidation. ACS Catal..

[43] Bataineh H., Pestovsky O., Bakac A. (2012). pH-induced mechanistic changeover from hydroxyl radicals to iron(iv) in the Fenton reaction. Chem. Sci..

[44] Wang X., Qin Y., Zhu L., Tang H. (2015). Nitrogen-doped reduced graphene oxide as a bifunctional material for removing bisphenols: Synergistic effect between adsorption and catalysis. Environ. Sci. Technol..

[45] Lu W., Xu T., Wang Y., Hu H., Li N., Jiang X., Chen W. (2016). Synergistic photocatalytic properties and mechanism of g-C_3_N_4_ coupled with zinc phthalocyanine catalyst under visible light irradiation. Appl. Catal. B Environ..

