# Supplementary material for: Dual-Mechanism Study of Metal-Free g-C3N4 Catalysts for Advanced Oxidation Under Non-Photocatalytic Conditions
Source: Molecules. 2025 Jan 10;30(2):247. doi: 10.3390/molecules30020247 (PMC11767740; doi:10.3390/molecules30020247)
Supplement: Supplementary file 1 [file molecules-30-00247-s001.zip › molecules-3381296-supplementary.pdf]

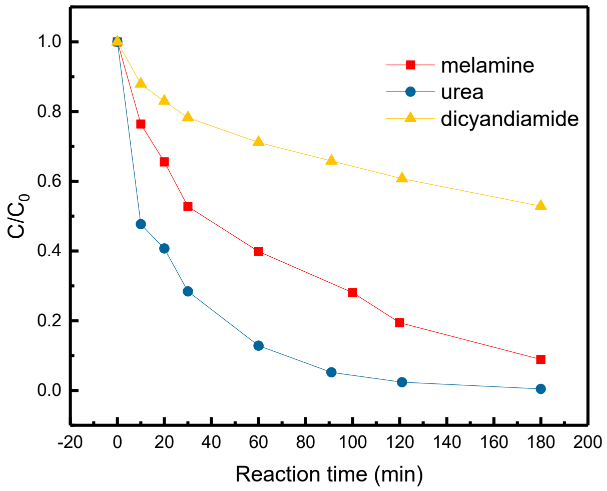


**Figure S1.** Degradation performance of g-$C_3N_4$ prepared with different precursor. (calcination program: 550 °C for 2 h; degradation condition: 5 ppm MB, 1000 ppm $H_2O_2$, 50 °C, 0.5g $L^{-1}$ catalyst)

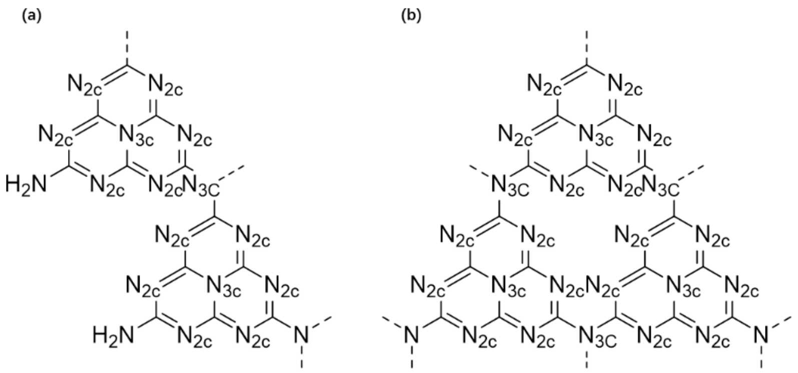


**Figure S2.** The molecular structures of g-$C_3N_4$ of different polymerization degree (a) partial condensation. (b) full condensation.
